# Basic Fibroblast Growth Factor Inhibits Apoptosis and Promotes Proliferation of Adipose-Derived Mesenchymal Stromal Cells Isolated from Patients with Type 2 Diabetes by Reducing Cellular Oxidative Stress

**DOI:** 10.1155/2017/3027109

**Published:** 2017-01-11

**Authors:** Daria Nawrocka, Katarzyna Kornicka, Joanna Szydlarska, Krzysztof Marycz

**Affiliations:** ^1^Department of Experimental Biology and Electron Microscope Facility, The Faculty of Biology and Animal Science, Wroclaw University of Environmental and Life Sciences, 50-631 Wroclaw, Poland; ^2^Wroclaw Research Centre EIT+, 54-066 Wroclaw, Poland

## Abstract

Type 2 diabetes (T2D) is a chronic metabolic disorder affecting increasing number of people in developed countries. Therefore new strategies for treatment of T2D and its complications are of special interest. Nowadays, cellular therapies involving mesenchymal stromal cells that reside in adipose tissue (ASCs) constitute a promising approach; however, there are still many obstacles concerning safety and effectiveness that need to be overcome before ASCs could be engaged for the treatment of diabetes mellitus. One of the challenges is preventing ASCs from deterioration caused by elevated oxidative stress present in diabetes milieu. In the current study we investigated the effect of basic fibroblast growth factor (bFGF) treatment on ASCs isolated from patients with diagnosed T2D. We demonstrate here that cell exposition to bFGF in 5 and 10 ng/mL dosages results in improved morphology, increased proliferative activity, reduced cellular senescence and apoptosis, and decreased oxidative stress, indicating recovery of ASCs' function impaired by T2D. Therefore our results provide a support for bFGF as a potential therapeutic agent for improving stem cell-based approaches for the treatment of diabetes mellitus and its complications.

## 1. Introduction

Over the past several decades World Health Organisation points out the fact that diabetes is becoming an increasing problem posing one of the most serious threats to global public health. Between 1980 and 2014 the number of adults diagnosed with diabetes has nearly quadrupled, accounting for 422 million people worldwide [1]. Predominant form of diabetes mellitus is type 2 (T2D), largely associated with obesity and lack of physical activity [2]. It comprises about 90–95% of all diabetic cases [3, 4]. The development of T2D involves metabolic abnormalities including insulin resistance in peripheral tissues, as well as impaired insulin synthesis and secretion due to disturbed *β*-cell function and loss of *β*-cell mass [5–7]. Current international guidelines for T2D treatment recommend the use of metformin insulin-sensitizer drug as the first-line medication, followed by the additional oral pharmacotherapy, and finally insulin supplementation [8, 9]. The weakness of this approach is that although these pharmacological agents can manage hyperglycemia or ameliorate response to insulin during their active use, they are not very effective in long-term overcoming of progressive loss of *β*-cell function and mass [10–12]. Alternative approach to diabetes therapy is *β*-cell replacement [13]. Islet transplantation is an experimental treatment directed to both type 1 and type 2 diabetic patients [14–16]. Benefits of the therapy include improved blood glucose control, reduction or even complete abolition of the need of exogenous insulin supply, and preventing hypoglycaemia [17]. However, this approach is fairly restricted by a shortage of primary human islets suitable for transplantation, troublesome immune rejection of the transplants, and financial barriers [18, 19]. Therefore, strategies for development of a new sources of insulin-producing cells for the treatment of diabetes have garnered significant interest. Recent studies have focused on self-renewable stem cells that due to their ability to differentiate into specialized cell types constitute a great source of replacement cells [20–22].

Human mesenchymal stromal cells (MSCs) have become an important population of progenitor cells with the potential to differentiate into adipocytes, chondrocytes, osteoblasts, and myocytes in vivo and in vitro when exposed to appropriate culture conditions [23, 24]. Due to their multipotent nature, capability to self-renew, and a capacity for modulation of immune responses, MSCs are considered as potential therapeutic agents for treatment of numerous diseases and regeneration of damaged tissues [25–27]. In diabetes research, MSCs have been involved to generate insulin-producing cells, suppress autoimmunity, and treat the complications of diabetes mellitus such as cardiomyopathy, nephropathy, retinopathy, polyneuropathy, and diabetic wounds [10, 28–30]. MSCs can be found in almost all organs and tissues of the body; thus it is fundamental to determine the preferable type or source of stem cells while developing a strategy for the therapy. Depending on the intended therapeutic application, MSCs can be isolated from, inter alia, bone marrow, umbilical cord blood, or adipose tissue [31, 32].

Given its abundance in the body and self-replenishing capability, adipose tissue stands as a great source of MSCs. It is easily accessible in large quantities and can be obtained during low-risk procedures such as liposuction; harvest is associated with minimal risk to the patients; and, finally, extraction of MSCs can be performed with high yield; therefore the need for cell expansion in culture is reduced [33, 34]. By the reason of all of those advantages, adipose tissue-derived mesenchymal stromal cells (ASCs) are enthusiastically used as research tools [35–37]. Nevertheless, to the best of our knowledge, the effect of diabetes on human ASCs has not yet been investigated; thus it is tempting to evaluate their properties and metabolic status in the context of diabetes treatment.

According to the former reports, diabetes causes the development of oxidative stress that is manifested by elevated intracellular reactive oxygen species (ROS) [38–41]. Thus, even though stem cells derived from patients with diabetes mellitus can be engaged for treatment of the disease and its complications, hyperglycemia-induced oxidative stress may compromise their proliferative potential and self-renewal ability and therefore influence the outcome of the stem cell-based therapy [42]. Our previous studies [43] stand in a good agreement with these findings, as we have demonstrated that ASCs isolated from horses suffering from EMS, metabolic disorder linked to insulin resistance, are characterized by altered morphology, senescent phenotype, loss of multipotency, decreased proliferative potential, and elevated oxidative stress. ASC_EMS_, when compared to cells isolated from healthy animals, exhibited higher *β*-galactosidase activity, increased rates of apoptosis, and reduced heterochromatin architecture underneath the nuclear envelope. Higher levels of nitric oxide (NO) and ROS, together with decreased superoxide dismutase (SOD) activity, were observed in these cells as well. Thus, deterioration of ASCs' function caused by diabetic microenvironment arouses concerns when clinical application for cellular therapy is considered. For this reason, different strategies to protect stem cell function and improve their therapeutic efficiency are being sought. One of the approaches involves conditioning in the presence of growth factors for enhancement of stem cell potency [42, 44].

In recent years, fibroblast growth factors (FGFs) and their receptors (FGFRs) were considered essential for embryonic development and tissue repair [45]. Basic fibroblast growth factor (bFGF) is a member of the FGF family that is alleged to promote self-renewal, maintain stemness, and suppress senescence of MSCs in vitro [45–48]. It has also been shown to enhance MSC osteogenic and chondrogenic differentiation [49, 50].

In the present study, effect of bFGF on human ASCs isolated from patients with type 2 diabetes was investigated. Our goal was to examine whether bFGF treatment can contribute to the reversal of the detrimental impact of diabetes mellitus on ASCs and therefore, in the future, improve clinical outcome of therapies involving those cells. We demonstrate here that the conditioning strategy based on cell exposition to bFGF results in enhanced proliferative activity, decreased cellular senescence and apoptosis, and reduced oxidative stress and thus in partially overcoming the ASCs' deterioration caused by T2D.

## 2. Materials and Methods

### 2.1. All Reagents Used in This Experiment Were Purchased from Sigma-Aldrich (Poland), Unless Indicated Otherwise

Cell handling and tissue processing procedures described here were conducted with the approval of the Local Bioethics Committee of Wroclaw Medical University (registry number KB-177/2014), in accordance with the ethical standards laid down in the 1964 Declaration of Helsinki and its later amendments.

### 2.2. ASC Isolation and Culture

Mesenchymal stromal cells were isolated from human subcutaneous adipose tissue as described previously by Grzesiak et al. [51] and Marycz et al. [52]. Adipose tissue fragments were harvested from adult healthy donors (age range: 60–76 years) or patients with type 2 diabetes (age range: 61–74) with written informed consent during total hip arthroplasty. ASCs collected from two different subjects for each sex were pooled within healthy and diabetic group to ensure that individual differences would not alter the results. In brief, the ASC isolation protocol was as follows: after extensive wash with Hank's Balanced Salt Solution (HBSS) supplemented with 1% antibiotic-antimycotic solution (Penicillin/Streptomycin/Amphotericin B, P/S/A), adipose tissue samples were minced mechanically and digested enzymatically with collagenase type I in a concentration of 1 mg/mL for 40 minutes at 37°C. Tissue homogenates were then clarified by centrifugation (1200 ×g, 10 minutes, room temperature). Supernatant was discarded, and cell suspension was subsequently washed with HBSS, centrifuged again, and resuspended in growth medium consisting of Dulbecco's Modified Eagle's Medium (DMEM) with Nutrient F-12 Ham, 10% of Fetal Bovine Serum (FBS), and 1% of P/S/A. Cells were seeded on polystyrene tissue culture flasks. Cultures were maintained at 37°C in a humidified atmosphere of 5% CO_2_ and 95% air. Medium was replaced every 2 days. Prior the experiment, the cells were passaged three times using trypsin solution after reaching 90% confluence (TrypLE™ Express, Life Technologies).

### 2.3. Immunophenotyping

The immunophenotypes of isolated cells were confirmed by flow cytometry. Human ASCs' surface antigen phenotyping was performed in a 1-step procedure using the following fluorophore-labelled monoclonal antibodies (mAbs): anti-CD34 phycoerythrin (PE), anti-CD45 PE, anti-CD90 fluorescein isothiocyanate (FITC), anti-CD105 pyridium-chlorophyll protein complex (PerCP), and anti-CD73b allophycocyanin (APC, all purchased from BD Pharmingen). For the analysis, human ASCs were detached from culture dishes using TrypLE Express solution, resuspended in Phosphate Buffer Saline (PBS), aliquoted, and stained with proper antibodies for 30 minutes at 4°C. At least 10,000 labelled cells were acquired and analysed using Becton Dickinson FACS Calibur apparatus. Data were processed using FlowJo software.

### 2.4. Multipotency Assay

Multilineage differentiation of the examined cell populations was assessed as described previously [53]. For the induction of adipo-, osteo-, and chondrogenesis, commercially available kits were used (StemPro, Life Technologies). Cultures under differentiation conditions were maintained in a 24-well plates at the initial concentration of 2 × 10^4^ cell/well. Adipogenesis was induced during 14-day period, while stimulation toward osteogenic and chondrogenic lineage lasted 21 days. All procedures were performed according to the manufacturer's instructions. Cultures expanded in standard growth medium provided control for the experiment and allowed establishing differentiation effectiveness. To evaluate the results of multilineage differentiation process, appropriate stainings were performed: Oil Red O to detect intracellular lipid droplets, Alizarin Red to visualize extracellular mineralized matrix, and Safranin O to confirm the formation of proteoglycans. Specimens were analysed using Axio Observer A1 inverted microscope (Zeiss, Germany). Documentation was made using Cannon PowerShot digital camera.

### 2.5. ASC Propagation with Basic Fibroblast Growth Factor (bFGF)

Prior the experiment, ASCs isolated from healthy or diabetic donors were seeded in 24-well plates in a concentration of 2 × 10^4^ cells/well and suspended in 1 mL of culture medium. The first dosage of bFGF was added to the culture after 24 hours, in order to allow the cells to attach to the plate surface before the stimulation. Two bFGF concentrations were tested: 5 ng/mL and 10 ng/mL. Reagent was added directly to the culture media. Untreated cells served as controls for comparison with the experimental groups. Media were changed every day along the 7-day experiment.

### 2.6. Cell Viability and Proliferation Assay

Cell proliferation rate was evaluated using a resazurin-based cytotoxic assay (In Vitro Toxicology Assay Kit, Sigma-Aldrich), following manufacturer's instruction. Briefly, culture media were collected and replaced with medium containing 10% of resazurin dye. The absorbance levels of the supernatants were measured using spectrophotometer (BMG Labtech, Germany) at a wavelength of 600 nm for resazurin and 690 nm reference length. Each measurement included a blank sample containing complete medium without cells. Resazurin bioreduction rate is proportional to the cell's viability and metabolic activity [54]. Cardinality of cells was determined after 24th, 72nd, and 120th hour of cell propagation on the basis of assay data, including standard curve.

Population doubling time (PDT) was calculated with the support of online software [55] based on the following formula:

(1) PDT=duration·log⁡2log⁡final concentration−log⁡initial concentration.

### 2.7. Colony Forming Unit-Fibroblastic Assay

To provide a measure to estimate ASCs' clonogenic potential, in vitro cell survival assay was performed. ASCs were seeded into 6-well plates in triplicate at a density of 100 cells/well in standard medium for 24 hours. The medium was then replaced and cultures were further maintained in the presence or absence of an appropriate dosage of bFGF. After 7 days, the cells were fixed with 4% ice-cold paraformaldehyde and stained with pararosaniline. Aggregates consisting of 50 or more cells were considered as colonies and visually scored as positive (Axio Observer A1 inverted microscope, Zeiss). The efficiency of colony forming (CFUfs) was calculated using the formula presented below, as described by Chen et al. [56]:

(2) $$\begin{array}{c} \text{CFUfs }\left[\;\%\;\right]=\frac{\text{the number of colonies}}{\text{initiall cell number}}\cdot 100\% \end{array}$$

### 2.8. Visualization of Cell Morphology

ASCs' morphology was evaluated under epifluorescent microscope (Zeiss, Axio Observer A1). Visualization of the actin cytoskeleton was performed using the immunofluorescence staining technique. Briefly, cells were rinsed in HBSS and fixed with 4% ice-cold paraformaldehyde overnight at 4°C. Thereafter, cells were washed extensively with HBSS and permeabilized for 15 minutes at room temperature with 0.2% Tween-20 in HBSS. Finally, cells were stained for 40 minutes in the dark at room temperature with atto-594-labelled phalloidin diluted 1 : 800 in HBSS to stain actin filaments. Nuclei were counterstained using diamidino-2-phenylindole (DAPI; 1 : 1000) for 5 minutes at room temperature. Photographs were acquired using a PowerShot camera (Cannon).

### 2.9. Evaluation of Cellular Senescence and Apoptosis Level

Senescence-associated beta-galactosidase (SA-*β*gal), a biomarker of senescent cells in culture, was detected by Senescence Cells Histochemical Staining Kit. Staining procedure was conducted in accordance with the manufacturer's instruction. Briefly, growth medium was removed from over cell monolayer; the cells were rinsed with PBS and then fixed with provided formaldehyde-based Fixation Buffer. After washing with PBS, the cells were incubated overnight with SA-*β*gal staining solution at 37°C without CO_2_.

Additionally, cell viability was assessed using a Cellstain Double Staining Kit. Combined fluorescent reagents were used to distinguish the populations of live and dead cells. Staining procedure was performed as described in the manufacturer's protocol. Viable cells were labelled with calcein-AM dye and emitted bright green fluorescence, whereas the nuclei of dead cells with compromised membranes were stained with propidium iodide (PI) and fluoresced red-orange. The samples were imaged using epifluorescent microscope (Axio Observer A1, Zeiss) and captured with Cannon PowerShot camera.

### 2.10. Evaluation of the Oxidative Stress Level and Antioxidative Capacity of ASCs

For the quantification of cellular oxidative stress, cell cultures were carried out in standard growth medium without phenol red for 7 days in the presence or absence of bFGF (5 or 10 ng/mL). Nitric oxide (NO) concentration was assessed with the Griess Reagent Kit, reactive oxygen species (ROS) with a H2DCF-DA solution (both purchased from Life Technologies). Antioxidant potential of ASCs was determined on the basis of superoxide dismutase (SOD) secretion levels detected by SOD Assay Kit. Additionally, total ROS in cells was detected by means of fluorescence microscopy, using Total ROS Detection Kit (Enzo Life Sciences). Furthermore, to determine whether mitochondria stand as a major source of ROS within the cells, cells were exposed to rhodamine-based dye, MitoRed, in 1 : 1000 dilution. All procedures were conducted following the protocols supplied by the manufacturers.

### 2.11. Immunocytochemistry

The procedure for direct immunofluorescent staining of cells in culture included the following steps: (i) cell fixation using 4% paraformaldehyde in PBS pH 7.4 for 30 minutes at room temperature; (ii) triple wash with HBSS, 5 minutes each; (iii) permeabilization with 0.5% Triton X-100 for 20 minutes at room temperature; (iv) extensive wash, as described above; (v) cell incubation with blocking buffer consisting of 10% Goat Serum and 0.2% Tween-20 in HBSS for 45 minutes to eliminate unspecific binding of the antibodies; (vi) overnight incubation at 4°C with primary antibodies directed against Ki-67, caspase-3 (both purchased from Abcam), GLUT-4, or FGFR-1 phosphorylated at Y653/Y654 (R&D Systems), diluted in HBSS containing 1% Goat Serum and 0.2% Tween-20; (vii) washing of cells (as described above); (viii) 1-hour incubation in the dark at room temperature with the secondary antibodies conjugated with atto-488 fluorescent label (1 : 100 dilution) followed by cell extensive rinsing with HBSS as previously described. Nuclei were visualized by DAPI counterstain (5 minutes at room temperature).

Visualization of 5-methylcytosine (5mC) and 5-hydroxymethylcytosine (5hmC) was performed as follows: (i) cell fixation with 4% paraformaldehyde; (ii) permeabilization using 0.5% Triton X-100 in HBSS; (iii) incubation with 4N HCl for 15 minutes at room temperature; (iv) 45-minute incubation with blocking buffer; (v) overnight incubation at 4°C with primary antibodies diluted 1 : 200 in HBSS supplemented with 1% Goat Serum and 0.2% Tween-20; (vi) extensive wash with HBSS; (vii) 1-hour incubation in the dark with secondary antibodies conjugated with atto-488 (5mC) or atto-594 (5hmC) diluted 1 : 100.

Samples were analysed using Axio Observer A1 inverted and epifluorescent microscope (Zeiss, Axio Observer A1), while the documentation was made using Cannon PowerShot camera.

### 2.12. Analysis of Gene Expression

Expression of selected genes was investigated by quantitative real-time reverse transcription polymerase chain reaction (qRT-PCR). Upon completion of the experiment, cells were rinsed twice with HBSS and subsequently homogenized using 1 mL of TRI Reagent. Total RNA isolation was performed by phenol-chloroform extraction, as described by Chomczynski and Sacchi [57]. Quantity and quality of totalRNA were assessed using a nanospectrophotometer at wavelengths 260/280 nm (WPA Biowave II). The synthesis of complementary DNA (cDNA) from RNA template via reverse transcription, together with genomic DNA digestion, was performed using PrimeScript™ RT Reagent Kit with gDNA Eraser (Perfect Real-Time, Takara Clontech). For each reaction 100 ng of totalRNA was used. Both procedures were performed in accordance with the manufacturer's instruction using a T100 Thermo Cycler (Bio-Rad). PCR reaction mixtures were prepared using a SensiFast SYBR and Fluorescein Kit (Bioline) and contained 2 *μ*L of cDNA in a total volume of 20 *μ*L. The final concentrations of primers were 500 nM. The reaction was performed using a CFX Connect™ Real-Time PCR Detection System (BioRad) and the following thermal cycling conditions: initial enzyme activation at 95°C for 4 minutes, followed by 50 cycles of 95°C for 15 seconds for denaturation, annealing temperature gradient for 30 seconds, and elongation at 72°C for 15 seconds with a single fluorescence measurement. The PCR primer sequences are listed in Table 1. Relative gene expression was calculated in relation to the GAPDH housekeeping gene.

### 2.13. Statistical Analysis

Each experiment was performed in triplicate. The data are reported as mean values ± standard deviations. Statistical significance was determined by one-way analysis of variance (ANOVA) with Tukey's post hoc multiple comparison test using GraphPad Prism 7 (San Diego, USA). Differences were considered significant at *p* value <0.05.

## 3. Results

### 3.1. Immunophenotype and Multipotent Properties of Isolated ASCs

**Figure 1:**
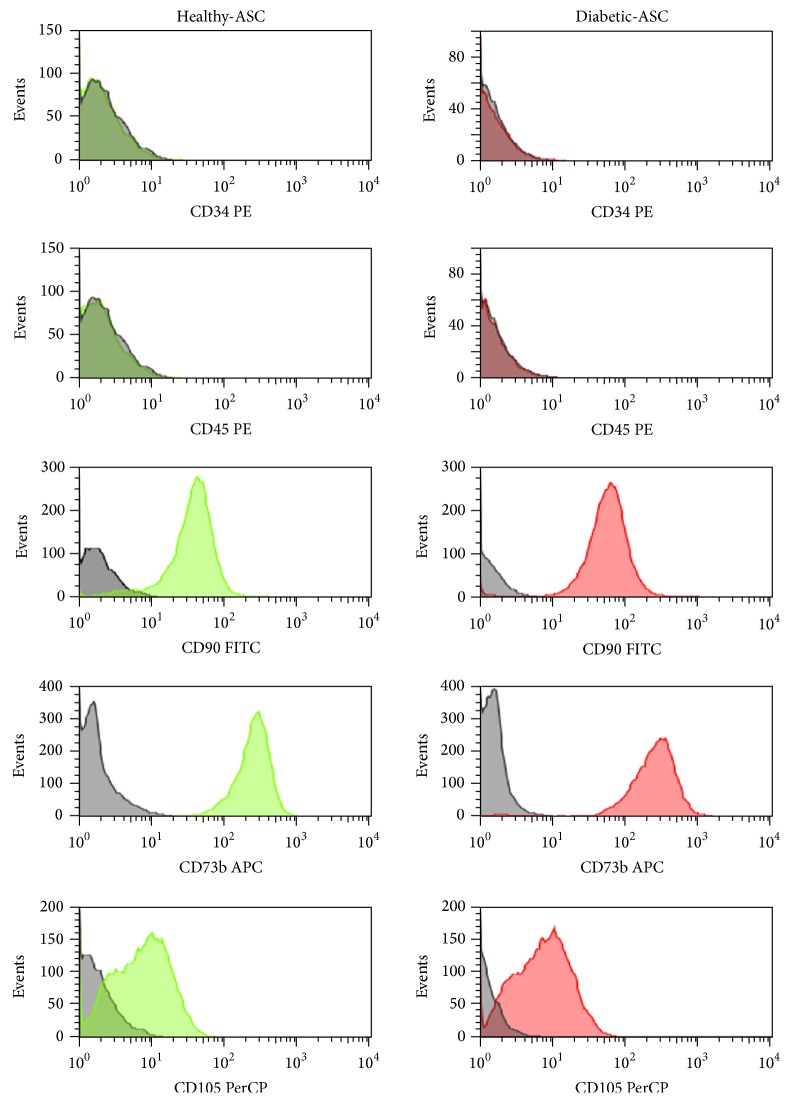
Characterization of healthy and diabetic-ASC phenotype by fluorescence-activated cell sorting (FACS). Passage 3 ASCs were analysed by flow cytometry after staining with fluorophore-labelled antibodies directed against indicated cell-surface proteins (green and red peaks). Unstained cells served as negative control for the analysis (grey peaks). Both ASCs isolated from healthy and type 2 diabetic donors expressed CD90, CD73b, and CD105 but were negative for CD34 and CD45 markers.

**Figure 2:**
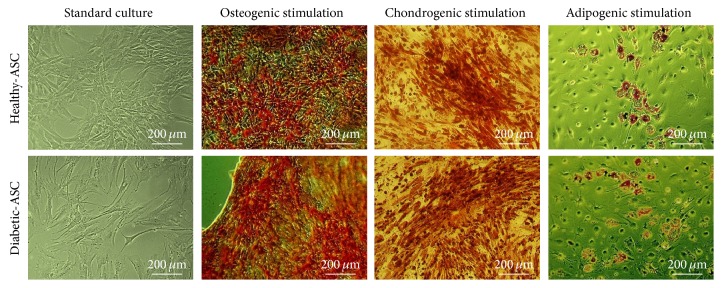
The morphology of ASCs derived from healthy or diabetic donors cultured in appropriate induction media. Lipid droplets accumulation in response to adipogenic stimulation was confirmed by Oil Red O staining, and mineral depositions in osteogenic cultures were detected with Alizarin Red, while cartilage formation following chondrogenic differentiation was evaluated using Safranin O reagent.

In order to characterize the ASCs according to the International Society for Cellular Therapy criteria for defining multipotent mesenchymal stromal cells [58], cell-surface marker expression was analysed by flow cytometry of ASCs derived from healthy and diabetic donors. Both cell populations displayed MSC-like antigen profile that exhibited high CD90, CD73b, and CD105 expression and lack of CD34 and CD45 hematopoietic markers (Figure 1). Additionally, multipotent nature of cells was confirmed by positive results of differentiation into osteoblast, chondrocytes, or adipocytes in vitro, as demonstrated by specific lineage staining (Figure 2).

### 3.2. Effect of Basic FGF on ASCs' Proliferation Activity and Clonogenic Potential

**Figure 3:**
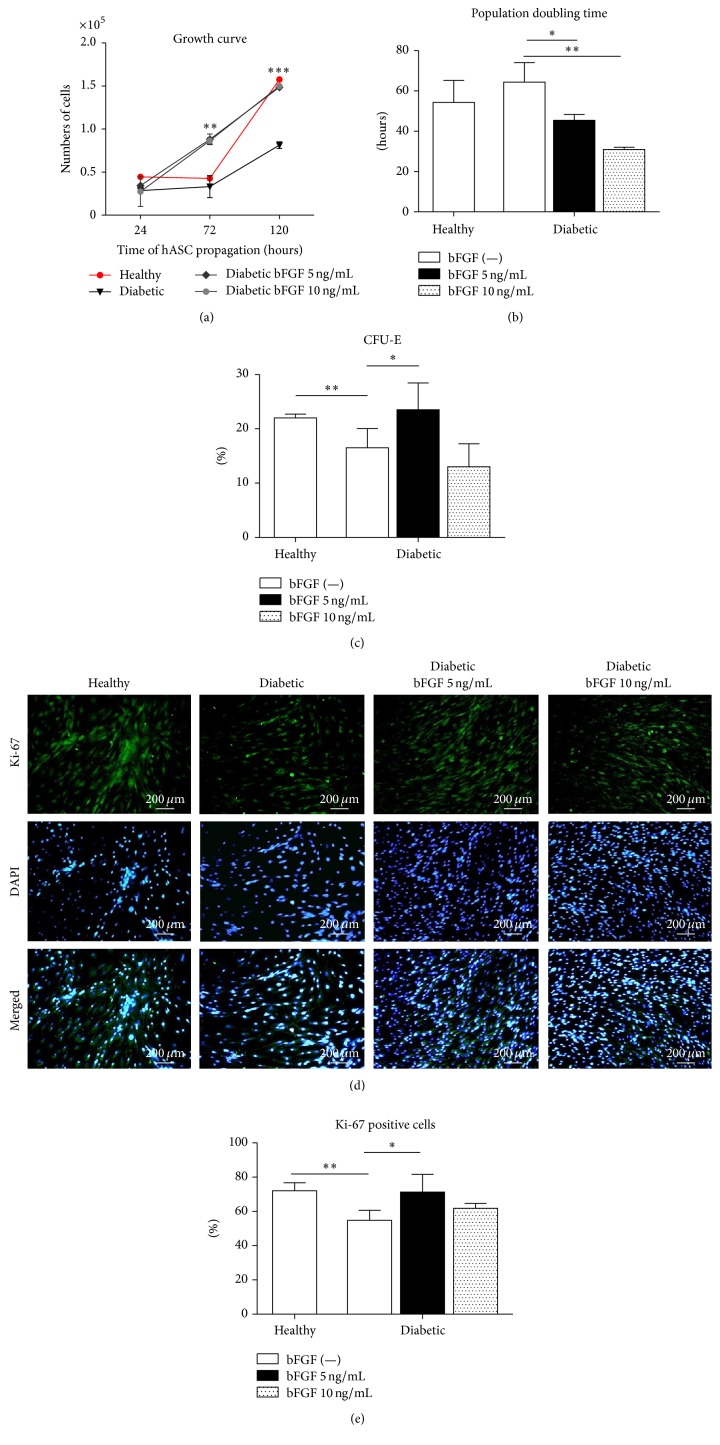
The effect of bFGF stimulation on ASCs' proliferative activity and clonogenic potential. Growth kinetics of diabetic-ASCs after bFGF treatment at concentrations of 5 ng/mL and 10 ng/mL in comparison to nontreated healthy and diabetic control ASCs (a). Supplementation of culture medium with bFGF resulted in restoration of reduced proliferation rate of diabetic-ASCs to the level of healthy-ASCs after 120 h of propagation. Population doubling time calculated after 120 h of cell propagation (b). bFGF-treated diabetic-ASCs were characterized by significantly abbreviated time required to double the population. CFU-E assay showing impaired clonogenic potential of diabetic-ASCs when compared to healthy-ASCs (c). Exposition to bFGF caused increase in the number of clonogenic precursors in diabetic-ASC cultures. Identification of proliferating cells by immunocytochemical staining for Ki-67 (d). Quantification of Ki-67 staining showing decreased percentage of Ki-67 expressing cells among diabetic-ASCs in respect to healthy-ASCs and increment in the number of proliferating cells following bFGF stimulation; fraction of Ki-67 positive cells was calculated on the basis of image analysis of 10 randomly selected pictures (e). Results are expressed as mean ± SD. ^*∗*^*p* value <0.05, ^*∗∗*^*p* value <0.01, and ^*∗∗∗*^*p* value <0.001.

In the first set of the experiments, we investigated whether bFGF induces a proliferative response in ASCs. The growth kinetics of ASCs in vitro, after exposition to the examined doses of bFGF, were evaluated after 24, 72, and 120 hours of culture (Figure 3(a)). Determination of cell proliferation activity in control cultures of ASCs derived from healthy (healthy-ASCs) or diabetic (diabetic-ASCs) donors revealed that the population remained stable for the first 72 hours, implying the lag phase. This was followed by a log phase in which the ASCs divided at exponential rates for the next 48 hours. However, growth rates of diabetic-ASCs were significantly slower and the number of cells generated by the end of 120 hours in culture was strongly reduced. Exposure of diabetic-ASCs to the bFGF accelerated growth of cells. The growth curves of experimental cultures had exponential character for the whole experiment. Reduced proliferation rate of ASCs from diabetic donors was increased after 72 hours of cell stimulation and almost completely recovered after 120 h. As shown in Figure 3(b), time required to double the population was significantly reduced for diabetic-ASCs cultured in the presence of bFGF at a concentration of 5 ng/mL (*p* < 0.05) and 10 ng/mL (*p* < 0.01).

The CFU-E assay results, presented in Figure 3(c), revealed significantly compromised colony forming efficiency of diabetic-ASCs as compared to those derived from healthy donors (*p* < 0.01). Increase in the number of clonogenic fibroblast precursor cells was apparent in culture treated with bFGF at concentration of 5 ng/mL (*p* < 0.05), but not in cells exposed to 10 ng/mL bFGF.

Proliferating cells were further identified after 7 days of the experiment by immunofluorescent antibody staining against Ki-67 (Figure 3(d)). Ki-67 intranuclear protein is widely used as a proliferation marker, as it can be detected during all active phases of cell cycle, but not in quiescent state [59]. The collected image data were quantitatively analysed for the percentage of Ki-67 expressing cells. The results are presented in Figure 3(e). As expected, the proportion of cells with nuclear Ki-67 immunoreactivity among diabetic-ASC population was significantly lower when compared with healthy-ASCs (*p* < 0.01). Stimulation of diabetic-ASCs with bFGF resulted in restoration of the number of proliferating cells, in case of 5 ng/mL concentration, to the level of healthy control culture. Increase in the number of Ki-67 positive cells was also observed in cultures exposed to 10 ng/mL bFGF; however, the difference between experimental and control group was not statistically significant.

### 3.3. The Morphology of ASCs Treated with Various Concentrations of bFGF

**Figure 4:**
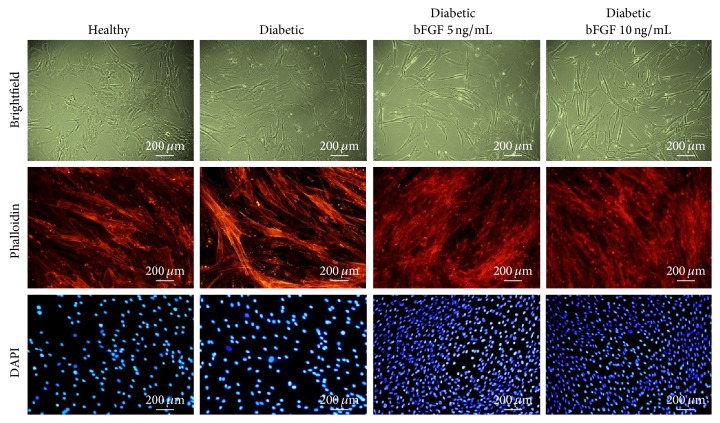
The morphology of healthy and diabetic-ASCs cultured in the presence or absence of bFGF in 5 ng/mL or 10 ng/mL concentration, evaluated after 7 days of cell propagation. Actin filaments were visualized with atto-594-labelled phalloidin and the nuclei with DAPI. Images included in the graph are representative and show characteristic features of examined cultures. In bFGF-treated cultures, the majority of cells exhibited elongated spindle-shaped morphology with no signs of apoptosis. Magnification: ×100.

Evaluation of the morphological changes in ASCs exposed to bFGF in reference to nontreated control cells harvested from diabetic or healthy donors was performed on the 7th day of the experiment (Figure 4). Healthy- and diabetic-ASCs maintained in the control conditions without bFGF supplementation exhibited various kinds of morphologies, including elongated bi- or multipolar fibroblast-like cells and flat spread-out cells of irregular shape with enlarged cytoplasm. The latter ones constituted the vast majority, especially in cultures of ASCs derived from diabetic patients. Both smaller fibroblast-like cells and large flat cells were characterized by centrally positioned nuclei and well-developed cytoskeleton. The cells closely adhered to each other and formed tight monolayers. The exposure to bFGF in a concentration of 5 or 10 ng/mL significantly influenced morphology and growth pattern of diabetic-ASCs. In bFGF-treated cultures, elongated spindle-shaped cells having a central nucleus, exhibiting no sign of apoptosis, were prevailed. Moreover, cells treated with bFGF grew more densely and after 7 days in culture formed multilayers, implying high proliferative activity. The confluence of bFGF-exposed diabetic-ASCs cultures was remarkably higher than control ones.

### 3.4. bFGF Suppresses Cellular Senescence and Apoptosis of Human ASCs Derived from Diabetic Patients

**Figure 5:**
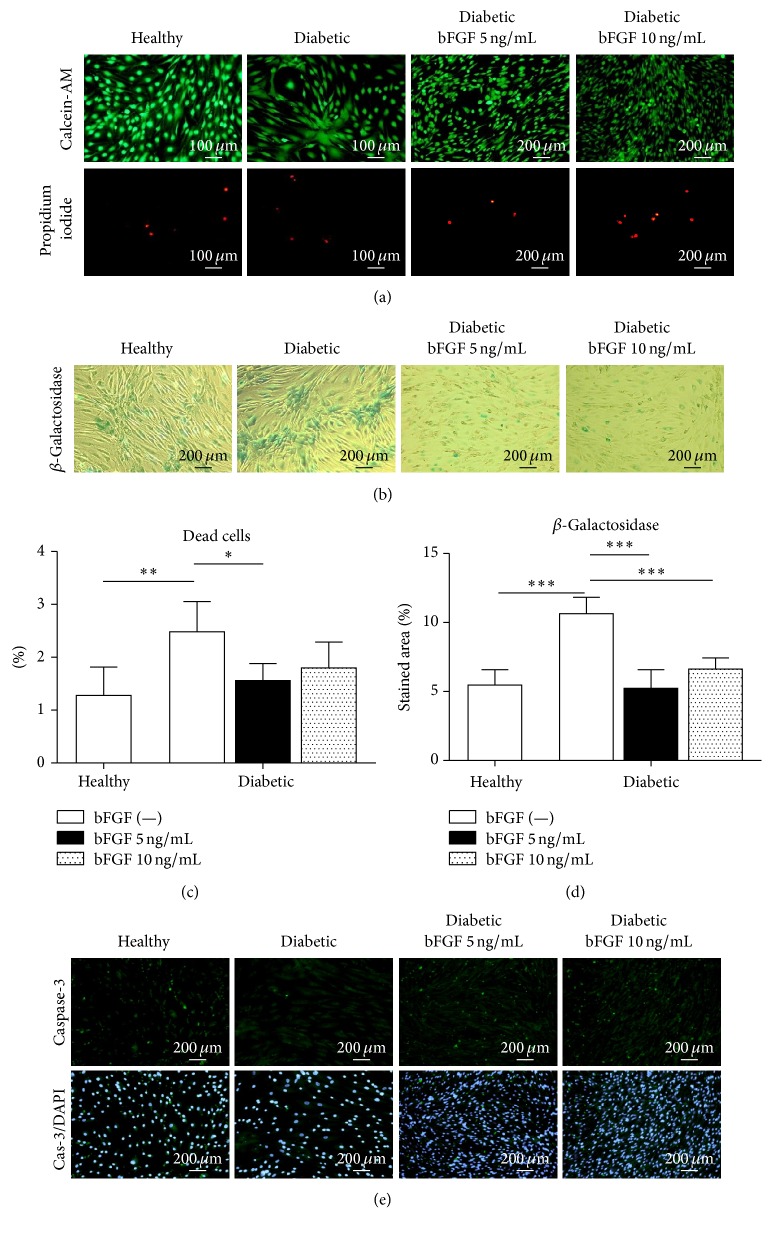
Cellular senescence and apoptosis level in healthy and diabetic-ASCs cultured under standard conditions or exposed to various doses of bFGF. Viable and dead cells were visualized by simultaneous fluorescent staining with calcein-AM and propidium iodide solutions, respectively (a). Senescent cells in cultures were indicated by detection of senescence-associated *β*-galactosidase (b). Proportion of dead cells was calculated on the basis of the image analysis of at least 10 representative shots of calcein-AM/propidium iodide double staining (c); similarly, the numerousness of senescent cells was determined by quantification of SA-*β*gal stained area (d). Additionally, apoptotic cells were identified by immunofluorescent staining of active caspase-3; nuclei were counterstained with DAPI (e). ^*∗*^*p* value <0.05, ^*∗∗*^*p* value <0.01, and ^*∗∗∗*^*p* value <0.001.

To verify, whether stimulation of diabetic-ASCs growth by bFGF in vitro is correlated with cellular senescence and apoptosis level, we determined the percentage of dead and senescent cells in investigated cultures. Calcein-AM and propidium iodide simultaneous fluorescence staining was performed to visualize viable and dead cells. Results are shown in Figure 5(a). On the basis of the image analysis, the percentage of dead cells in each experimental group was calculated (Figure 5(c)). The amount of apoptotic cells in diabetic-ASC culture was notably elevated when referred to the healthy-ASCs (*p* < 0.01). In cultures treated with bFGF, the ratio was moved toward living cells, especially when stimulant in a concentration of 5 ng/mL was used, in which case the difference regarding untreated diabetic-ASC was statistically significant (*p* < 0.05). In line with those results are data obtained from the analysis of senescence-associated *β*-galactosidase accumulation (Figure 5(b)). Senescent cells expressing SA-*β*gal were detected with intended staining kit. Resulting images were additionally analysed in terms of the percentage of stained area using ImageJ software to provide quantitative data (Figure 5(d)). The results clearly demonstrate that numerousness of senescent cells in ASC cultures established from diabetic patients is increased when compared to the cultures originating from healthy donors (*p* < 0.001). A sufficient decrease (*p* < 0.001) in the expansiveness of stained area was observed in cultures exposed to bFGF. The inhibition of ASCs cellular senescence by bFGF occurred in a dose-independent manner. In turn, the expression of caspase-3 in cells grown in growth medium supplemented with bFGF was more abundant than in cells grown in growth medium alone. There were only substantial differences in the quality and intensity of reaction between ASCs derived from healthy and diabetic donors (Figure 5(e)).

### 3.5. Quantitative Analysis of Apoptosis-Related Gene Expression

**Figure 6:**
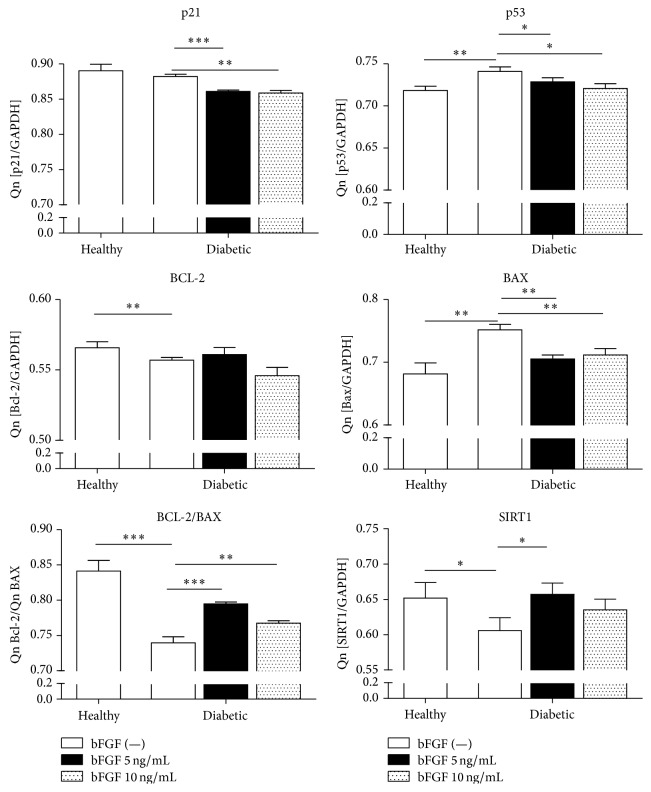
Apoptosis-related gene expression. Real-time RT-PCR analysis of the expression of p21, p53, Bcl-2, Bax, and SIRT1 mRNA in ASCs derived from healthy or diabetic donors, cultured in the presence or absence of bFGF for 7 days. Additionally Bcl-2/Bax mRNA expression ratio was calculated, as it negatively correlates with the size of apoptosis. ^*∗*^*p* value <0.05, ^*∗∗*^*p* value <0.01, and ^*∗∗∗*^*p* value <0.001.

In the next step of the study we assessed SIRT1, p21, p53, Bax, and Bcl-2 mRNA levels using real-time RT-PCR, and after that we evaluated the quantitative relation between Bax, apoptosis promoter, and Bcl-2, apoptosis inhibitor (Figure 6). Analysis of the expression of p21 and p53, well known apoptosis-inducing genes, revealed significant upregulation of p53 (*p* > 0.01), but not p21, in diabetic-ASCs. Moreover, Bcl-2/Bax ratio calculated for this group was strongly diminished (*p* < 0.001), implying susceptibility of ASCs derived from diabetic patients to apoptosis. The amount of p21 transcript was significantly reduced in cells treated with bFGF in a dose of 5 ng/mL (*p* < 0.001) and 10 ng/mL (*p* < 0.05). Similarly, p53 gene expression was suppressed in cells exposed to bFGF (*p* < 0.05 irrespective of the dose). Consistently, Bcl-2/Bax ratio was remarkably lower in cells grown in the presence of 5 ng/mL (*p* < 0.001) or 10 ng/mL (*p* < 0.01) bFGF. Additionally, the effect of bFGF treatment on sirtuin 1 gene expression in diabetic-ASCs was evaluated. SIRT1 is a NAD^+^-dependent deacetylase that targets a variety of proteins, including those known of controlling apoptosis, cell defences, and metabolism [60, 61]. SIRT1 has been shown to deacetylate and thus inhibit, inter alia, p53 protein [62]. Quantitative analysis of transcripts revealed that the expression of SIRT1 in ASCs harvested from diabetic patients was notably decreased in comparison to the ASCs isolated from healthy donors (*p* < 0.05). The propagation of cells in the presence of 5 ng/mL bFGF caused significant upregulation of SIRT1 mRNA expression (*p* < 0.05). SIRT1 mRNA level was likewise elevated in diabetic-ASCs stimulated with bFGF in 10 ng/mL concentration; however the difference with respect to the nonstimulated cells was not statistically significant. Altogether, our data suggest that bFGF may contribute to suppression of cellular apoptosis in diabetic-ASCs.

### 3.6. bFGF Stimulation Reduces Oxidative Stress in ASCs Isolated from Diabetic Donors

**Figure 7:**
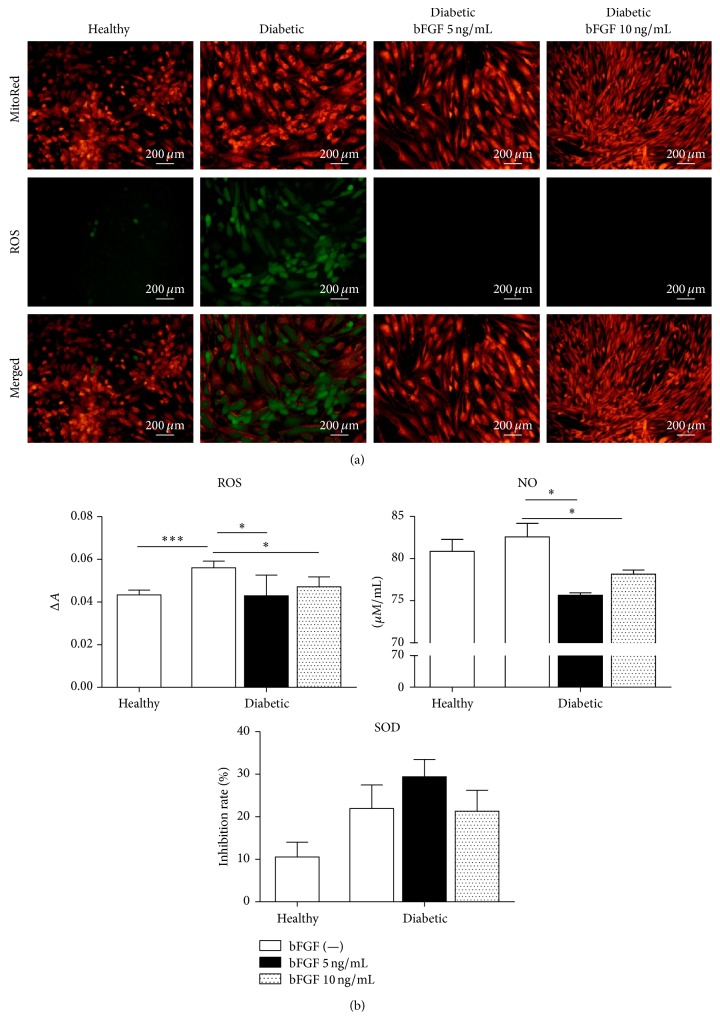
Changes in oxidative stress level in diabetic-ASCs after exposition to bFGF in a concentration of 5 ng/mL or 10 ng/mL with reference to nontreated healthy and diabetic-ASC controls. Detection of intracellular ROS along with mitochondria localization via MitoRed stain in investigated cultures by fluorescence microscopy (a). Quantification of extracellular ROS, NO, and SOD levels (b). ^*∗*^*p* value <0.05 and ^*∗∗∗*^*p* value <0.001.

To examine whether bFGF improves stem cell function by overcoming altered redox state in ASCs derived from diabetic patients, we measured the intracellular levels of reactive oxygen species (ROS) in investigated cultures. Fluorescence microscopy showed that ROS production in diabetic-ASCs was abundantly increased when compared with ASCs derived from nondiabetic donors (Figure 7(a)). Interestingly, intracellular ROS was completely alleviated in cells exposed to bFGF in both tested concentrations (5 and 10 ng/mL). Additionally, to ascertain whether mitochondria are involved in generation of ROS, we used the MitoRed mitochondrial stain. Comparative analysis of both stainings revealed that ROS subcellular localization overlaps with that of MitoRed. This indicates that mitochondria are a major source of ROS in studied cells.

Owing to the fact that oxidative stress factors either accumulate within cells or can be released into the culture media [63, 64], we examined the latter ones for ROS and nitric oxide (NO) levels. Moreover, to evaluate cellular antioxidant activity, we quantified extracellular superoxide dismutase (SOD) secretion. All measurements were performed using spectroscopic methods. Results are presented in Figure 7(b). In compliance with data from intracellular ROS staining, extracellular ROS levels were markedly augmented in cultures of diabetic-ASCs when compared to control cells from healthy patients (*p* < 0.001). A slight increase in excreted NO was as well evident in this group; however, the difference between diabetic-ASC and healthy-ASC cultures was not statistically significant. ROS and so too NO secretion was relevantly downregulated upon treatment of diabetic-ASCs with bFGF. Response rates were similar regardless dose of the bFGF (*p* < 0.05). Bafflingly, measurement of secretory extracellular SOD revealed increased activity of this antioxidant in diabetic-ASCs collating to the healthy-ASC cultures. Stimulation of cells with bFGF in 5 ng/mL concentration resulted in minor upregulation of SOD secretion; nevertheless, the differences between control and experimental groups were not statistically significant.

### 3.7. Detection of FGFR-1 mRNA and Receptor Phosphorylation in ASCs

**Figure 8:**
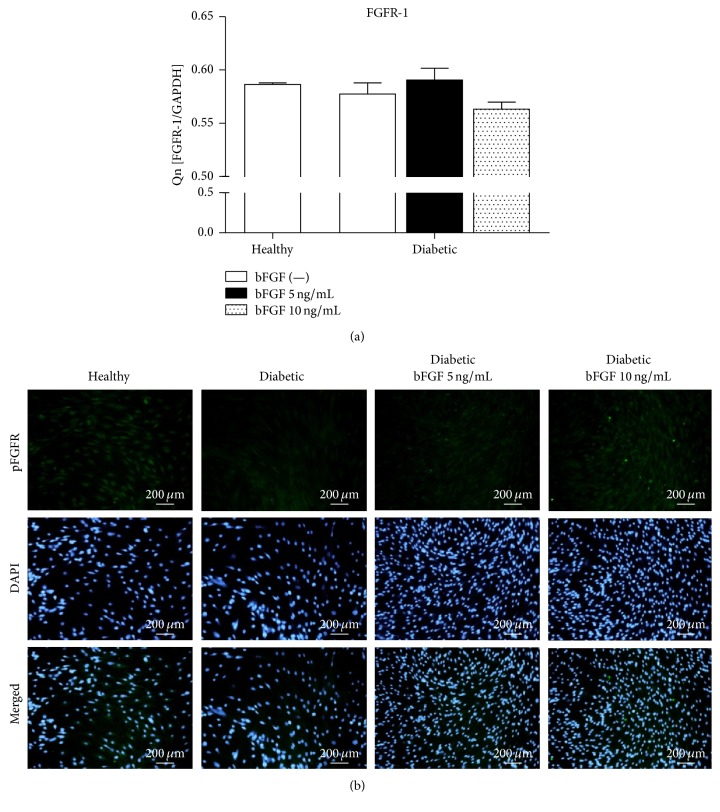
Analysis of FGFR-1 mRNA and phosphorylated receptor levels in investigated ASCs. FGFR-1 gene expression was evaluated by real-time RT-PCR (a). Activated FGFR-1 phosphorylated at Y653/Y654 was additionally detected with the use of anti-phospho-FGFR-1 antibody; nuclei were counterstained with DAPI (b). Magnification: ×100.

The next stage of our research was evaluation of fibroblast growth factor receptor 1 (FGFR-1) gene expression. FGFR-1 is a receptor tyrosine kinase whose ligand is inter alia basic FGF [65]. Upon stimulation with bFGF, the receptor undergoes tyrosine autophosphorylation. Subsequently, DNA synthesis is stimulated and proliferative response is induced in cells expressing the receptor [66, 67]. Analysis of FGFR-1 mRNA levels revealed no significant differences between investigated groups (Figure 8(a)). The amounts of the transcript were comparable in both diabetic-ASCs and healthy controls. Exposition of cells to bFGF also did not affect considerably the receptor expression in diabetic-ASCs. However, detection of activated receptor with the use of anti-phospho-FGFR-1 (Y653/Y654) antibody demonstrated markedly reduced pFGFR-1 in diabetic-ASCs (Figure 8(b)). Treatment of diabetic-ASCs with bFGF resulted in increased pFGFR-1 protein levels. The size of the increase was proportional to the concentration of the reagent.

### 3.8. Regulation of GLUT-4 Glucose Transporter Expression in ASCs by bFGF

**Figure 9:**
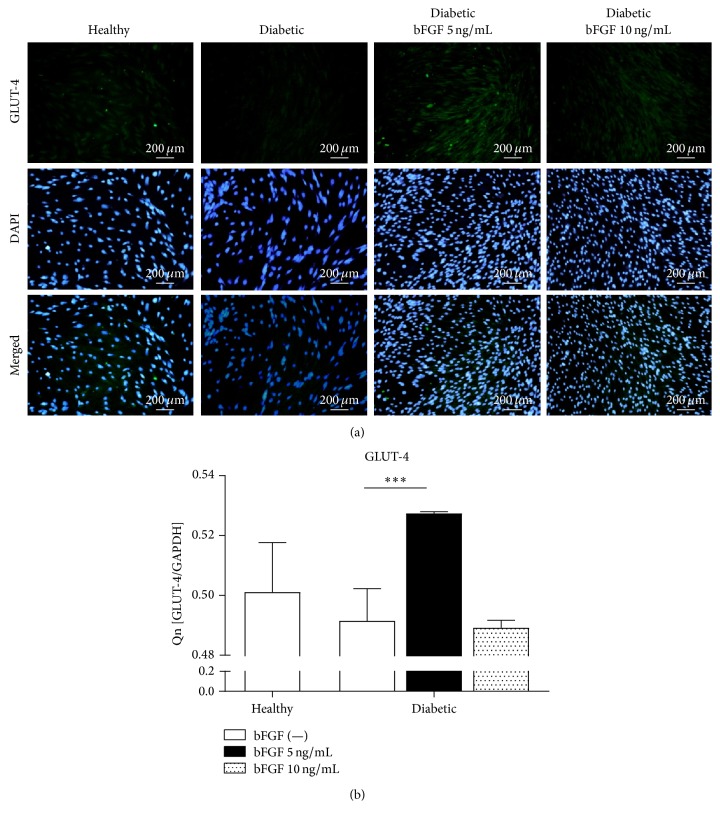
Evaluation of GLUT-4 mRNA and protein levels in healthy and diabetic-ASCs cultured under standard conditions or in the presence of investigated bFGF concentrations. GLUT-4 protein was detected in examined cultures by immunofluorescent staining, magnification ×100 (a). Expression of GLUT-4 gene was quantified by real-time RT-PCR (b). ^*∗∗∗*^*p* value <0.001.

To investigate the effect of bFGF on regulating glucose metabolism, we detected glucose transporter isoform GLUT-4 in ASCs by immunofluorescence staining. Results are presented in Figure 9(a). GLUT-4 density in ASCs from diabetic patients was markedly reduced as compared to the healthy control group. Stimulation of cells with bFGF in a concentration of 5 ng/mL and, to a minor extent, 10 ng/mL contributed to restored expression of GLUT-4 protein. Moreover, additional analysis of GLUT-4 gene expression by the means of real-time RT-PCR (Figure 9(b)) revealed significantly augmented transcript level (*p* < 0.001) following exposition to bFGF in a concentration of 5 ng/mL. Interestingly, increasing the dose of bFGF to 10 ng/mL resulted in abolition of this effect. There was no statistical difference in the GLUT-4 mRNA level neither between the group treated with bFGF in 10 ng/mL concentration and diabetic control group nor between healthy and diabetic-ASCs maintained in control conditions.

### 3.9. The Effect of bFGF Treatment on Genome-Wide 5mC and 5hmC Distribution and TET Gene Expression

**Figure 10:**
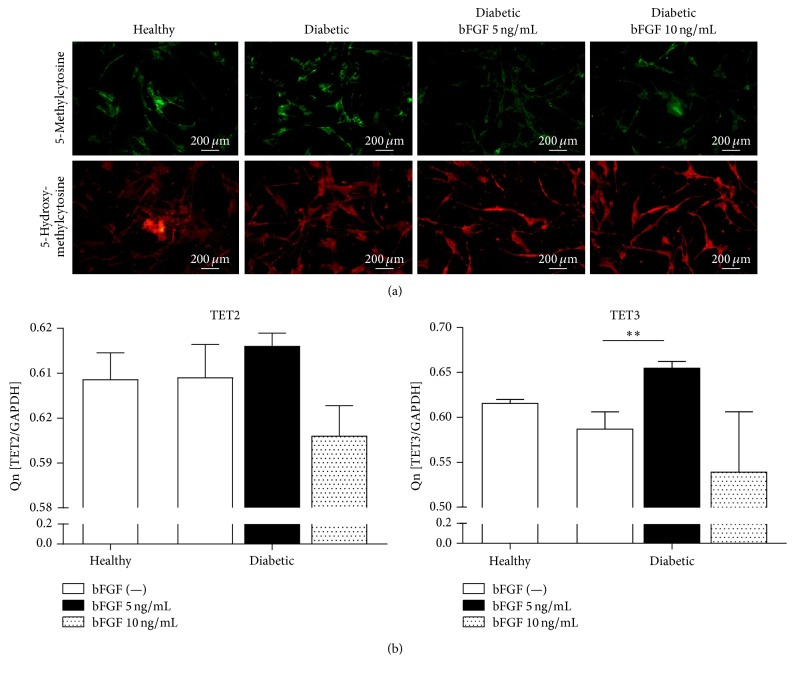
Evaluation of DNA methylation status in examined ASCs. Distribution of genome-wide 5-mC and 5-hmC was assessed by immunofluorescent staining after 7 days of culture in the presence or absence of bFGF, magnification ×100 (a). The expression of TET2 and TET3 methylcytosine dioxygenases was analysed by real-time RT-PCR (b). ^*∗∗*^*p* value <0.01.

To evaluate whether bFGF stimulation affects DNA methylation status, the genomic distribution of 5mC and 5hmC was determined by immunofluorescence staining after 7 days of culture in the presence or absence of the stimulant (Figure 10(a)). The results show that the overall distribution pattern of investigated molecules in healthy- and diabetic-ASCs is quite similar. There was no appreciable difference in global 5-mC and 5-hmC levels between both groups; however, a considerable decline of 5mC along with increase of 5hmC was observed in diabetic-ASCs subjected to stimulation with bFGF, especially when 5 ng/mL dose was applied. To investigate whether reduction of 5mC correlates with augmented TET2 and TET3 methylcytosine dioxygenases expression, we assessed the levels of both transcripts in examined groups by real-time RT-PCR (Figure 10(b)). We found that the expression of both TET2 and TET3 was increased in diabetic-ASCs after exposition to bFGF in 5 ng/mL concentration. The difference with respect to the same cells maintained in the control conditions was statistically significant in case of TET3 gene (*p* < 0.01). In contrast, propagation of diabetic-ASCs with 10 ng/mL bFGF caused slight inhibition of TET2 and TET3 mRNA expression; however the decrease was not statistically significant. There were no significant differences in TET2 and TET3 mRNA levels between ASCs derived from healthy and diabetic donors.

## 4. Discussion

Mesenchymal stromal cells with their multidifferentiation capability, self-renewal potential, and powerful immunomodulatory effect constitute attractive therapeutic agents for various disorders, including diabetes and its complications [30, 68–71]. A readily accessible source of multipotent stem cells is adipose tissue. Ease of availability and expansion makes adipose tissue-derived stem cells (ASCs) interesting approach for cellular therapies and tissue engineering. Unfortunately, there are still many obstacles concerning safety and effectiveness that need to be overcome before ASCs could be engaged for the treatment of diabetes mellitus [72, 73]. A large body of evidence suggests the presence of systemic oxidative stress in obese insulin-resistant subjects, causing abnormalities in the adipose tissue and leading to attenuation of ASCs' regenerative potential [74–76]. Therefore strategies for improving the quality and efficacy of ASCs isolated from diabetic donors are of special interest for researchers.

The principal objective of this research endeavour was to investigate whether conditioning strategy based on basic fibroblast growth factor treatment could be successfully applied to cope with adverse impact of oxidative stress milieu on ASCs derived from patients with type 2 diabetes.

bFGF has been widely reported as multipotent cytokine that stimulates proliferation of mesenchymal stromal cells originating from a variety of sources [77–86]. Analysing data obtained by, among others, Sotiropoulou et al. [80], Solchaga et al. [81, 82], Kim et al. [83], Wu et al. [84], and Wang et al. [86], we have chosen two bFGF concentrations as likely optimal for treatment of human ASCs expanded as monolayers, that is, 5 ng/mL and 10 ng/mL. Our results are in line with our expectations and published reports showing recovery of impaired proliferative activity of ASCs derived from diabetic donors and dose-dependent decrease in population doubling time. Furthermore, the effect of bFGF on ability of mesenchymal stromal cells to form colonies has been previously evaluated by Walsh et al. [87] and Wu et al. [84]. The study of Walsh et al. [87] demonstrated that treatment with bFGF in a dose of 2.5 ng/mL has no effect on colony formation efficiency of bone marrow stromal cells, but it does increase the size of the colonies. On the other hand Wu et al. [84] reported that stimulation of stem cells from the apical papilla with bFGF in 5 ng/mL concentration results in improved colony formation and escalated colonies' size. Current research showed enhanced clonogenic potential of diabetic-ASCs exposed to bFGF in 5 ng/mL concentration; nonetheless this effect was lost, when the dose of the stimuli was increased to 10 ng/mL. The acceleration of cell growth and improved colony formation of diabetic-ASCs treated with bFGF correlated with augmentation of Ki-67 cellular proliferation marker levels. Ki-67 is a nuclear protein that can be detected during all active phases of cell cycle (G_1_, S, G_2_, and M) but is absent in the resting phase (G_0_) of quiescent cells [88]. The upregulation of Ki-67 expression resulting from bFGF treatment was previously noted in mesenchymal stromal cells and described by Coutu et al. [89], suggesting that bFGF signalling may be involved in regulating senescence in MSCs in vitro. These findings are also consistent with the results of SA-*β*gal staining and calcein-AM/propidium iodide viability assay. The percentage of senescent and dead cells was markedly decreased in cultures propagated with bFGF, insinuating bFGF-induced resistance to apoptosis in diabetic-ASCs. Paper of Coutu et al. [89] supported the thesis that bFGF promotes MSC proliferation mainly by inhibiting cellular senescence through PI3K/AKT–MDM2 signalling pathway; therefore, it can play a key role in maintenance of MSCs' self-renewal and stemness. Interestingly, our study demonstrates that the reduced number of PI-positive and SA-*β*gal expressing cells in bFGF-treated cultures does not go together with lessened tendency to form apoptotic bodies. The abundance of caspase-3 positive cells was comparable whether cultures were maintained in the presence or absence of bFGF. At first glance, this phenomenon may seem quite remote from the primary effect of bFGF on cellular survival; it was, notwithstanding, previously discussed in literature. Schamberger and her group [90] observed that the addition of bFGF rescued rat 423-cells from apoptosis induced by serum withdrawal and that this process tended to advance downstream caspase-3 activation.

bFGF affected physiology of diabetes-impaired ASCs not only by ameliorating proliferative activity and viability of those cells, but also by causing morphological alterations. Epifluorescence microscopy analysis revealed that ASCs originating from diabetic patients developed more fibroblast-like elongated phenotype and achieved higher cell densities when exposed to both tested dosages of bFGF. This finding complies with studies of other research groups, like Guillot et al. [91], who described morphology of adult bone marrow-derived MSCs cultured in the presence of bFGF (5 ng/mL) as fibroblastic with spindle-shaped cytoplasm of reduced volume in regard to control cells expanded in the absence of bFGF, or Kabiri et al. [92] demonstrating that supplementation of growth medium with bFGF in a concentration of 10 ng/mL results in smaller cytoplasmic volume and spindle-shape morphotype of cultured human adipose-derived adult stem cells. Similar observations were made by Martin et al. [93], who have showed that BMSC cultured with bFGF were thinner, more elongated, and, in contrast to the control cultures which gained more flattened phenotype, maintained original fibroblastic shape in the following passages. They concluded that this phenomenon might be explained by either direct effect of bFGF on MSCs' differentiation or bFGF regulation of synthesis and organisation of extracellular matrix, which would lead to altered cell spreading pattern and in consequence phenotype.

To get insight into molecular mechanism underlying bFGF-mediated rescue from senescence and prevention of apoptosis in diabetic-ASCs, we analysed the expression of p21, p53, Bcl-2, Bax, and SIRT1 mRNAs. p21 and p53 are widely considered as apoptosis-inducing genes. Many previous studies have suggested that p53/p21 pathway is involved in regulating MSCs' senescence progress [94–99] and proliferation [99]. Moreover, it has been shown that p21 expression is associated with abnormal proliferation, colony formation, and apoptosis of BM-MSCs from the nonobese diabetic mice [100]. Some other reports indicate that diabetes-induced hyperglycemia accelerates endothelial progenitor cells' senescence through the activation of p38/MAPK and Akt/p53/p21 pathways [101–103] and downregulation of sirtuin 1 [104]. In the ASCs from patients with type 2 diabetes, we observed upregulated p21 and p53 genes. This finding is compatible with our previous study, showing increased p21 and p53 mRNA levels in ASCs originating from EMS horses [43]. Exposition of diabetic-ASCs to bFGF in 5 or 10 ng/mL concentrations resulted in significant inhibition of both p21 and p53 expression. Obtained data stand in good agreement with the findings of Ito et al. [46] who reported that in vitro stimulation of human MSCs growth with bFGF results in decreased percentage of senescent cells in culture and suppressed G1 cell cycle arrest through the inhibition of p21, p53, and p16 expression levels. Furthermore, we have found that bFGF treatment represses elevated Bax levels in diabetic-ASCs. Proapoptotic Bcl-2 family member Bax resides primarily inactive in cytosol and translocates to mitochondria upon induction of apoptosis to release apoptogenic factors [105]. During apoptosis, Bax typically induces loss of the electrochemical gradient (ΔΨ) across the inner mitochondrial membrane and opens the voltage-dependent anion channel (VDAC) allowing cytochrome c to pass through the channel. Once released from the mitochondria, cytochrome c binds to the APAF-1 and initiates caspases activation. Upon those events apoptotic protease cascade is triggered and programmed cell death occurs. While Bax promotes the loss of ΔΨ, cytochrome c release, and activation of caspases, overexpression of Bcl-2 in cells inhibits those changes prolonging cell survival [106–108]. Furthermore, there is a large body of evidence that the expression of Bax and Bcl-2 is regulated by p53 tumour suppressor gene and has been shown to be involved in p53-mediated apoptosis [109]. Therefore we investigated how bFGF treatment affects Bcl-2/Bax ratio, which is useful prognostic marker, as the balance between pro- and antiapoptotic members of Bcl-2 family can determine the cellular fate [110]. We found out that bFGF stimulation resulted in increased Bcl-2/Bax ratio in diabetic-ASCs indicating enhanced cell survival and diminished apoptotic response to metabolic stress. Moreover, we have also observed upregulation of SIRT1 after exposure of diabetic-ASCs to bFGF. Sirtuin 1 is a NAD-dependent deacetylase that has been shown to deacetylate p53 tumour suppressor protein and modulate p53 dependent functions correlated with oxidative stress responses, cellular metabolism, aging process, and apoptosis [62, 111]. Activation of SIRT1 by FGF family members has been formerly described in literature. Chau et al. have demonstrated that FGF21 regulates mitochondrial activity and enhances oxidative capacity in adipocytes through AMPK–SIRT1–PGC1*α* pathway [112].

Impaired morphology, proliferative potential, senescence, and apoptosis in ASCs originating from patients suffering from type 2 diabetes may be interrelated with elevated oxidative stress in diabetic milieu. Oxidative stress, defined as imbalance between the production of reactive oxygen species (ROS) and/or reactive nitrogen species (RNS) and protective mechanisms of antioxidant defence, has been reported to affect MSCs' longevity and functions, as well as reducing their ex vivo expansion leading to major repercussion on their clinical application [113]. Oxygen related free radicals (O_2_^−∙^, OH^∙^) and reactive species (H_2_O_2_, NO, and ONOO^−^) are primarily produced as a result of aerobic metabolism and under steady state are maintained at low, nontoxic concentrations by a variety of antioxidant defences, including superoxide dismutase (SOD). However, when released in excess in response to specific stress conditions, oxidative stress factors determine various pathological processes leading in consequence to tissue damage and, eventually, cell death [114–116]. Moreover, it is widely acknowledged that oxidative stress contributes to p53-mediated cell cycle arrest, DNA damage, and apoptosis [117]. Zhao and his group [118] published that p53 upon ROS activation migrates to mitochondria, where it interacts with manganese superoxide dismutase reducing thereby its superoxide scavenging activity and causing subsequent decline in mitochondrial membrane potential. p53 translocates then to the nucleus, where initiation of its transcriptional activity on targets genes such as Bax occurs enforcing mechanisms of p53-induced apoptosis. In turn, Passos et al. demonstrated that extended activation of p21 checkpoint gene triggered mitochondrial dysfunction and formation of ROS through GADD45-p38(MAPK14)-GRB2-TGFBR2-TGF*β* signalling pathway [119]. Our research showed that bFGF treatment effectively suppressed elevated intracellular mitochondrial ROS as well as extracellular ROS and NO released into the culture media of ASCs derived from diabetic patient. In addition, slightly increased level of secreted SOD was detected in cells treated with bFGF in 5 ng/mL concentration; however, it is worth pointing out that, to our surprise, differences in SOD activity between investigated groups were not statistically significant. Nevertheless, in accordance with the referenced reports [118, 119], suppression of oxidative stress by bFGF correlated with decreased transcripts levels of p21 and p53 genes in bFGF-treated diabetic-ASCs.

GLUT-4 is well studied glucose transporter highly expressed in adipose tissue and skeletal muscle. It plays a major role in whole body glucose homeostasis mediating the uptake of glucose regulated by insulin [120]. A great many previous studies have correlated reduced GLUT-4 gene and protein expression to the insulin-resistant glucose transport in adipocytes from obese and diabetic subjects [121–125]. These observations lead to the conclusion that upregulating GLUT-4 might be effective approach for the treatment of type 2 diabetes. Herein we showed that exposition of mesenchymal stromal cells isolated from adipose tissue of patients with type 2 diabetes to bFGF in a dose of 5 ng/mL caused significant increase in the level of GLUT-4 gene and protein expression, suggesting enhanced insulin sensitivity of those cells. In addition, reduction of global DNA methylation levels in diabetic-ASCs in response to 5 ng/mL bFGF was observed. These data further support insulin-sensitizing functions of bFGF at that particular concentration, as the association of altered global DNA hypermethylation with increased risk of insulin resistance was evidenced [126, 127].

## 5. Conclusions

In summary, the current study revealed that supplementation of culture media with bFGF has powerful effect on adipose-derived mesenchymal stromal cells originating from patients with type 2 diabetes. bFGF-induced recovery of diabetic-ASCs function was manifested by improved morphology, increased cellular proliferation rate, enhanced clonogenic potential, decreased senescence and apoptosis, and, finally, augmented insulin sensitivity. Demonstrated results suggest that beneficial action of bFGF results from overcoming adverse impact of oxidative stress milieu. The most pronounced effect was observed after application of bFGF in a dose of 5 ng/mL.

## Figures and Tables

**Table 1:** Sequences of primers used in real-time RT-PCR.

Gene	Sequence 5′-3′	Amplicon length (bp)	Accession number
p21	F: AGAAGAGGCTGGTGGCTATTT	169	NM_001220777.1
	R: CCCGCCATTAGCGCATCAC		
p53	F: AGATAGCGATGGTCTGGC	381	NM_001126118.1
	R: TTGGGCAGTGCTCGCTTAGT		
Bcl-2	F: ATCGCCCTGTGGATGACTGAG	129	NM_000633.2
	R: CAGCCAGGAGAAATCAAACAGAGG		
Bax	F: ACCAAGAAGCTGAGCGAGTGTC	365	XM_011527191.1
	R: ACAAAGATGGTCACGGTCTGCC		
SIRT1	F: ACAGGTTGCGGGAATCCAAA	155	NM_001314049.1
	R: GTTCATCAGCTGGGCACCTA		
FGFR-1	F: CCGCCCAACTTTTCCTCCAA	511	XM_006716314.1
	R: AGGTGGCATAACGGACCTTG		
GLUT-4	F: CTCTGAGTTGAGGGCAAGGG	3734	XM_011523750.1
	R: GGGGGACCTACCGCAACATA		
TET2	F: GAGACGCTGAGGAAATACGG	258	NM_001127208.2
	R: TGGTGCCATAGGAGTGGACA		
TET3	F: CAGACCGCTGTGATCGTCAT	263	NM_001287491.1
	R: AACTTGCGAGGTGTCTTGCT		
GAPDH	F: GTCAGTGGTGGACCTGACCT	256	NM_001289746.1
	R: CACCACCCTGTTGCTGTAGC		

F: sense primer; R: antisense primer; bp: base pair.
