# Corrigendum to “Basic Fibroblast Growth Factor Inhibits Apoptosis and Promotes Proliferation of Adipose-Derived Mesenchymal Stromal Cells Isolated from Patients with Type 2 Diabetes by Reducing Cellular Oxidative Stress”

**DOI:** 10.1155/2017/1083618

**Published:** 2017-04-22

**Authors:** Daria Nawrocka, Katarzyna Kornicka, Joanna Szydlarska, Krzysztof Marycz

**Affiliations:** ^1^Department of Experimental Biology and Electron Microscope Facility, The Faculty of Biology and Animal Science, Wroclaw University of Environmental and Life Sciences, 50-631 Wroclaw, Poland; ^2^Wroclaw Research Centre EIT+, 54-066 Wroclaw, Poland

In the article titled “Basic Fibroblast Growth Factor Inhibits Apoptosis and Promotes Proliferation of Adipose-Derived Mesenchymal Stromal Cells Isolated from Patients with Type 2 Diabetes by Reducing Cellular Oxidative Stress” [1], an acknowledgment should be added as follows:

“Publication was supported by the Wrocław Centre of Biotechnology programme, the Leading National Research Centre (KNOW) for years 2014–2018.”
